# Supplementary material for: A pH-sensitive closed-loop nanomachine to control hyperexcitability at the single neuron level
Source: Nat Commun. 2024 Jul 4;15:5609. doi: 10.1038/s41467-024-49941-3 (PMC11224301; doi:10.1038/s41467-024-49941-3)
Supplement: Supplementary file 1 — Supplementary information [file 41467_2024_49941_MOESM1_ESM.pdf]

NCOMMS-23-46298A
2nd Revised Version

# A pH-sensitive closed-loop nanomachine to control hyperexcitability at the single neuron level

Assunta Merolla[1,2]*, Caterina Michetti[1,3]*, Matteo Moschetta[1,2], Francesca Vacca[1], Lorenzo Ciano[1,3], Laura Emionite[2], Simonetta Astigiano[2], Alessandra Romei[1], Simone Horenkamp[1], Ken Berglund[4], Robert E. Gross[4], Fabrizia Cesca[1,5§], Elisabetta Colombo[1,2§], Fabio Benfenati[1,2]

[1] Center for Synaptic Neuroscience and Technology, Istituto Italiano di Tecnologia, Genova, Italy
[2] IRCCS Ospedale Policlinico San Martino, Genova, Italy
[3] Department of Experimental Medicine, University of Genova, Italy
[4] Department of Neurosurgery, Emory University School of Medicine, Atlanta, GA, USA
[5] Department of Life Sciences, University of Trieste, 34127 Trieste, Italy

*Equal contribution
§ Corresponding authors

# SUPPLEMENTARY INFORMATION

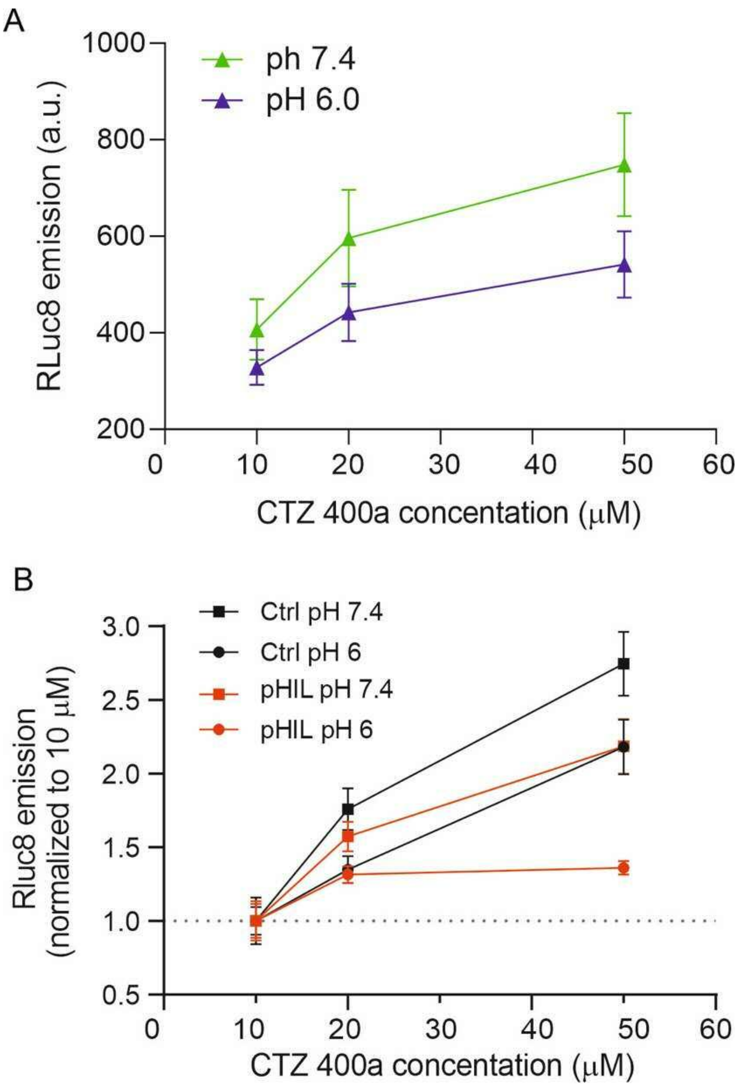


**Supplementary Figure 1. Effects of CTZ400a concentration on the emission of RLuc8 and $E^2$GFP at neutral and acidic pH. A.** Mean (± SEM) RLuc8 emission from HEK293 cells transfected with RLuc8 after the addition of increasing concentrations of the substrate CTZ 400a at pH 6.0 and 7.4. **B.** Mean (± SEM) RLuc8 emission from HEK293 cells transfected with either Ctrl (black line and symbols) or pHIL (red line and symbols) plasmids after the addition of increasing concentrations of CTZ400a at pH 6.0 and 7.0. Data were normalized to the mean emission at lower CTZ400a concentration (n = 6 independent cell preparations).

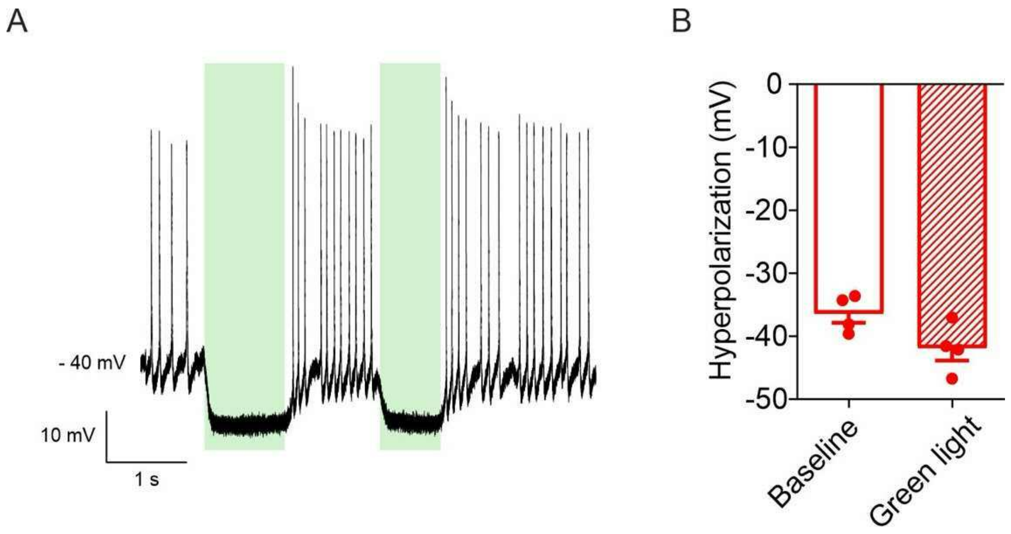


**Supplementary Figure 2. Direct excitation of pHIL-incorporated NpHR causes hyperpolarization and firing silencing in primary neurons. A.** Representative trace of current-clamp recordings of primary hippocampal neurons expressing pHIL upon direct excitation of NpHR (illumination at 530 nm, 30 mW/mm$^2$). **B.** Mean (± SEM) hyperpolarization of the membrane potential in response to direct activation of NpHR in pHIL-expressing neurons (n = 4 cells).

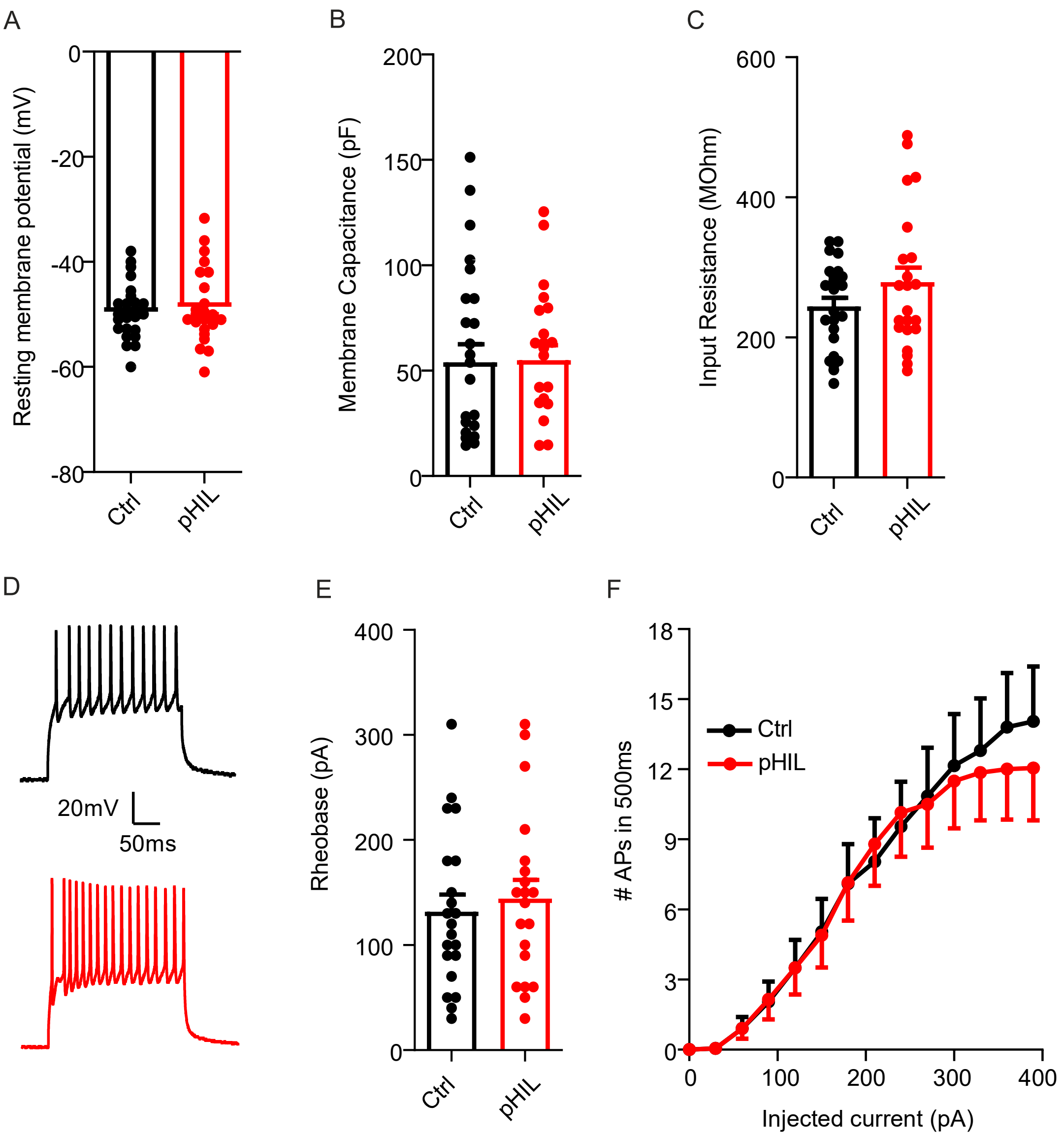


**Supplementary Figure 3. Expression of pHIL in primary hippocampal neurons does not alter their passive and active electrophysiological properties. A-C.** Resting membrane potential, cell capacitance and input resistance show no interference of neither construct with the passive properties of the transduced neurons (Resting membrane potential, n = 24, 25; Membrane capacitance, n = 22, 25; Input resistance, n = 22, 24 for Ctrl and pHIL, respectively). **D.** Representative whole-cell current clamp traces of evoked action potentials (APs) in primary hippocampal neurons expressing either Ctrl (black) or pHIL (red). **E.** The current intensity needed to reach the firing threshold (rheobase) is not affected by transduction of neurons with either Ctrl or pHIL (n = 20, 21 for Ctrl and pHIL, respectively). **F.** The frequency of APs evoked by the injection of increasing current steps is unaffected by the expression of either Ctrl or pHIL in primary neurons (n = 20 for both Ctrl and pHIL). Numerosity (n) refers to neurons derived from 2 independent preparations. Data in panels B-D are means ± SEM.

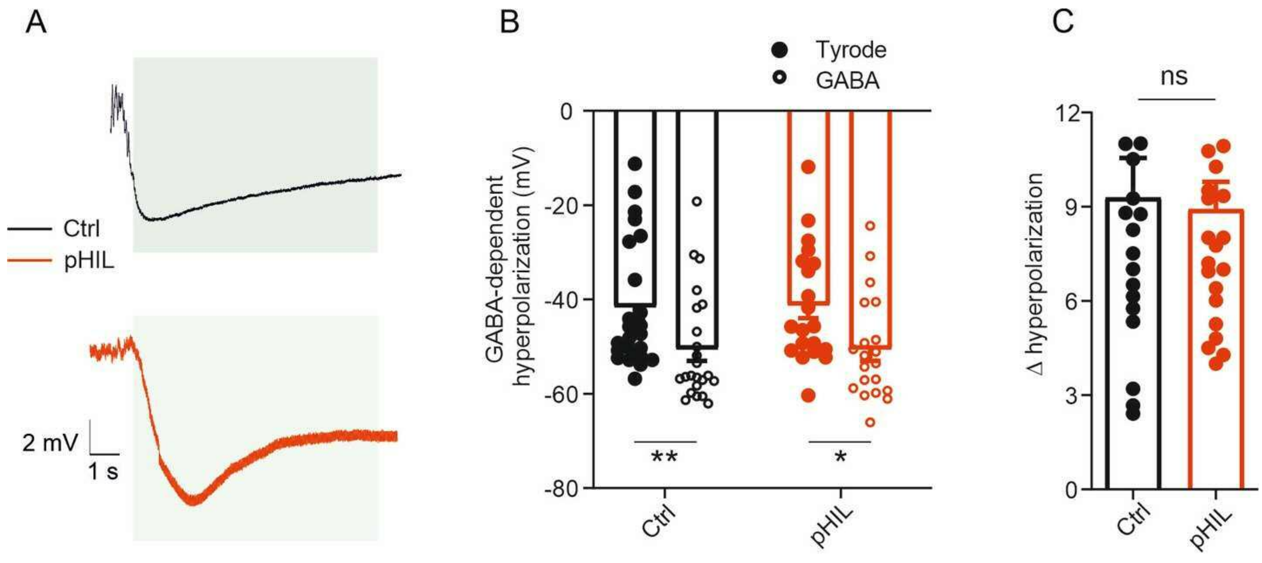


**Supplementary Figure 4. Expression of pHIL in primary hippocampal neurons does not affect GABA-induced hyperpolarization. A.** Representative traces of whole-cell current clamp experiments in primary hippocampal neurons transduced with either Ctrl (black) or pHIL (red) upon treatment with exogenous GABA (100 μM, grey area). **B.** GABA induces a significant hyperpolarization in both Ctrl- and pHIL-expressing neurons with respect to baseline in Tyrode (n = 23, 20 neurons for Tyrode and GABA treatment, respectively for both Ctrl and pHIL from 2 independent preparations, two-sided Student's *t*-test/Mann-Whitney's *U*-test; *p=0.0153, **p=0.0044). **C.** No significant difference in GABA-induced hyperpolarization was found in Ctrl- and pHIL-treated neurons. Data are means ± SEM (two-sided Mann-Whitney's *U*-test; ns, not significant).

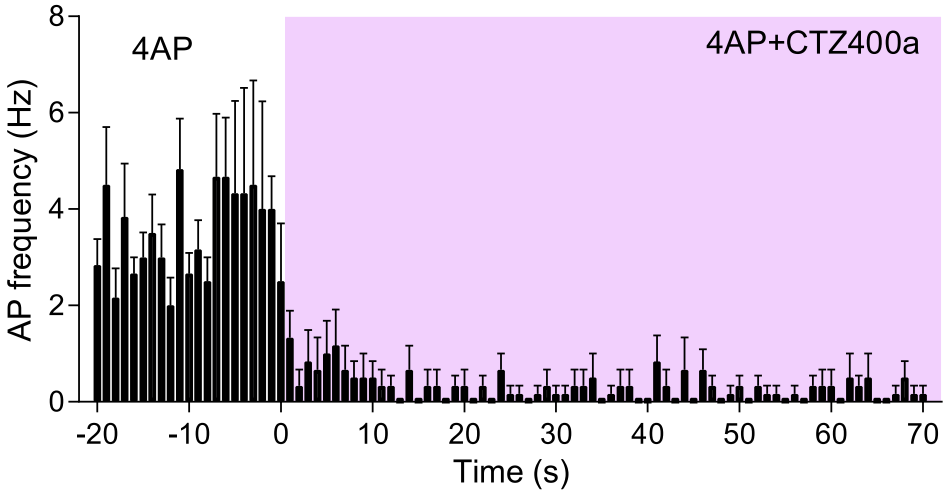


**Supplementary Figure 5. Time-course of the effect of CTZ400a administration on the paroxysmal activity of primary hippocampal neurons induced by 4AP.** Primary hippocampal neurons transduced at 7 DIV with pHIL and subjected at 14 DIV to treatment with the $K^+$ channel blocker 4AP (100 µM) starting at time - 20 s. After full expression of hyperexcitability, coelenterazine 400a (CTZ400a) was added to trigger endogenous light emission (purple area). Activation of pHIL induced a prompt decrease of AP firing that persisted for the whole recording period (70 s, n = 6 neurons from 2 independent preparations).

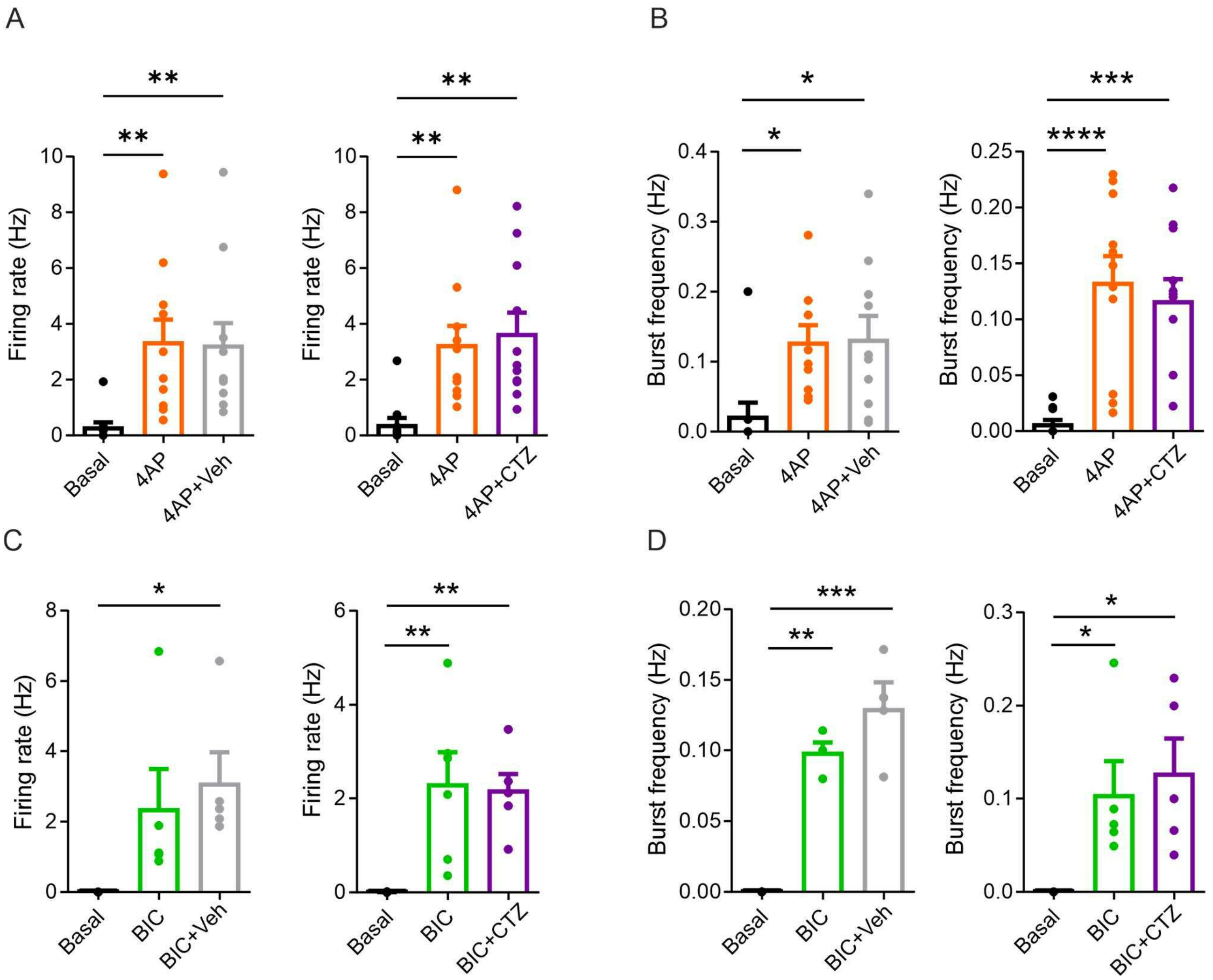


**Supplementary Figure 6. The Ctrl construct does not interfere with pharmacologically induced hyperactivity in primary hippocampal neurons.** Cell-attached voltage-clamp recordings from primary hippocampal neurons transduced with AAV2/1-Ctrl in Tyrode solution (basal), after treatment with either 4AP (**A,B**; 100 μM) or BIC (**C,D**; 30 μM) and after the subsequent addition of either CTZ400a (CTZ; 40 μM) or vehicle (Veh; ethanol 100%/2-Hydroxypropyl-β-cyclodextrin mixture). The analysis of the mean ± SEM firing rate (**A,C**) and bursting frequency (**B,D**) reveal the expected increase after the administration of either convulsant, which in both cases is not affected by the subsequent infusion of CTZ (firing rate: n = 11, Friedman's test, p=0.0004, Friedman statistic=13.82, Dunn's multiple comparisons test, **p=0.0085 (Basal vs 4AP), **p=0.0019 (Basal vs 4AP+Veh); n = 11 p=0.0004, Friedman statistic=13.82, Dunn's multiple comparisons test **p=0.0019 (Basal vs 4AP), **p=0.0085 (Basal vs 4AP+CTZ); for 4AP+Veh and 4AP+CTZ, respectively; n = 5, one-way repeated measures ANOVA F(2,8)=7.342, p=0.0155, Tukey's multiple comparisons test, *p=0.0156 and n = 6, one-way repeated measures ANOVA F(2,10)=13.05 p=0.0016, Tukey's multiple comparisons test **p=0.0028 (Basal vs BIC), **p=0.0041 (Basal vs BIC+CTZ), for BIC+Veh and BIC+CTZ, respectively; bursting rate: n = 11, Friedman's tests p=0.0075, Friedman statistic=9.6, Dunn's multiple comparisons test *p=0.0219 for both comparisons and n = 11, one-way repeated measures ANOVA F(2,20)=20.10 p<0,0001, Tukey's adjusted p values ***p=0.0002, ****p<0.0001 for 4AP+Veh and 4AP+CTZ, respectively; one-way repeated measures ANOVA F(2,6)=30.42 p=0.0007, Tukey's multiple comparisons test **p=0.0031, ***p=0.0007 and n = 6, one-way repeated measures ANOVA F(2,8)=8.423 p=0.0107, Tukey's multiple comparisons test *p=0.0323 (Basal vs BIC), *p=0.012 (Basal vs BIC+CTZ) for BIC+Veh and BIC+CTZ, respectively).

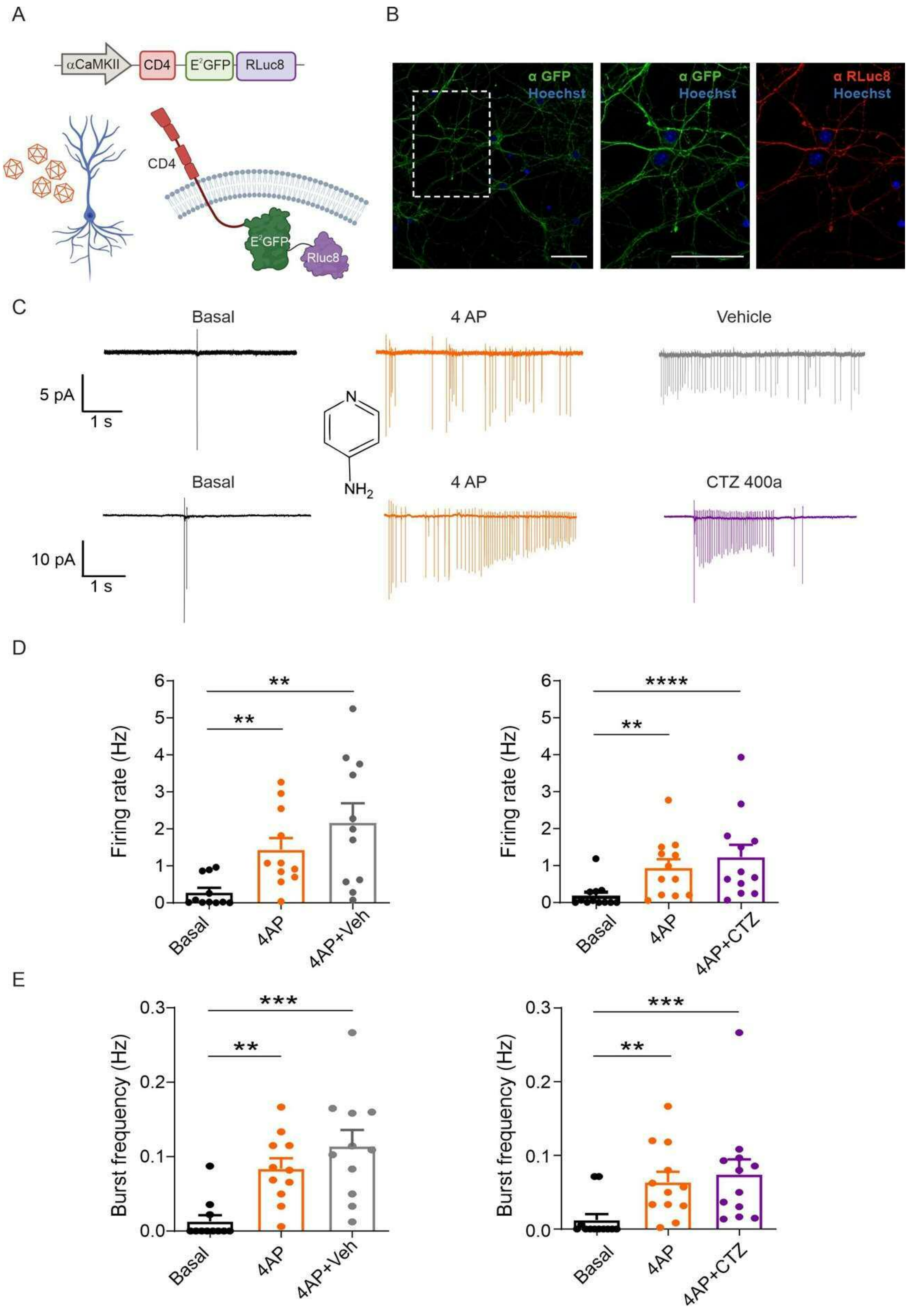


**Supplementary Figure 7. A control construct with a functionally silent transmembrane component does not affect 4AP-induced neuronal hyperactivity in response to CTZ400a. A.** Schematics of the AAV2/1 vector encoding Ctrl-CD4 (CD4-E$^2$GFP-Rluc8) expressed under the control of the CaMKIIα promoter and representation of the synthetic Ctrl-CD4 structure and cell membrane topology (Created with BioRender.com released under a Creative Commons Attribution-NonCommercial-NoDerivs 4.0 International license (https://creativecommons.org/licenses/by-nc-

nd/4.0/deed.en). **B.** *Left panel:* Representative confocal image of the membrane localization of Ctrl-CD4 in primary hippocampal neurons showing $E^2$GFP (green) and Rluc8 (red) immunoreactivities in the presence of Hoechst nuclear staining (blue). *Middle and right panels:* higher magnification of the boxed area shows that Ctrl-CD4 decorates the membranes of soma and neurites. Scale bars, 100 µm. **C.** Cell-attached voltage-clamp traces from Ctrl-CD4 transduced primary hippocampal neurons incubated in Tyrode solution (basal) and after the sequential additions of 4AP (100 µM) and either CTZ400a (CTZ; 40 µM) or vehicle (Veh; ethanol 100%/2-Hydroxypropyl-β-cyclodextrin mixture). **D,E.** The mean (± SEM) firing rate (**D**) and bursting frequency (**E**) of Ctrl-CD4 transduced neurons treated with 4AP show the hyperexcitability induced by the convulsant, which is not affected by the subsequent infusion of either vehicle or CTZ (firing rate: n = 11, one-way repeated measures ANOVA F(1.697,16.97)=10.84 p=0.0014, Tukey's multiple comparisons test **p=0.0099, **p=0.0078; n = 12, p<0.0001, Friedman statistic=19.5, Dunn's multiple comparisons test **p=0.0085, ***p=0.0002 for 4AP+Veh and 4AP+CTZ, respectively; bursting frequency: p<0.0001, Friedman statistic=17.64, Dunn's multiple comparisons test **p=0.0066, ****p<0.0001 and p<0.0001, Friedman statistic=18.67, Dunn's multiple comparisons test **p=0.0033, ***p=0.0001 for 4AP+Veh and 4AP+CTZ, respectively).

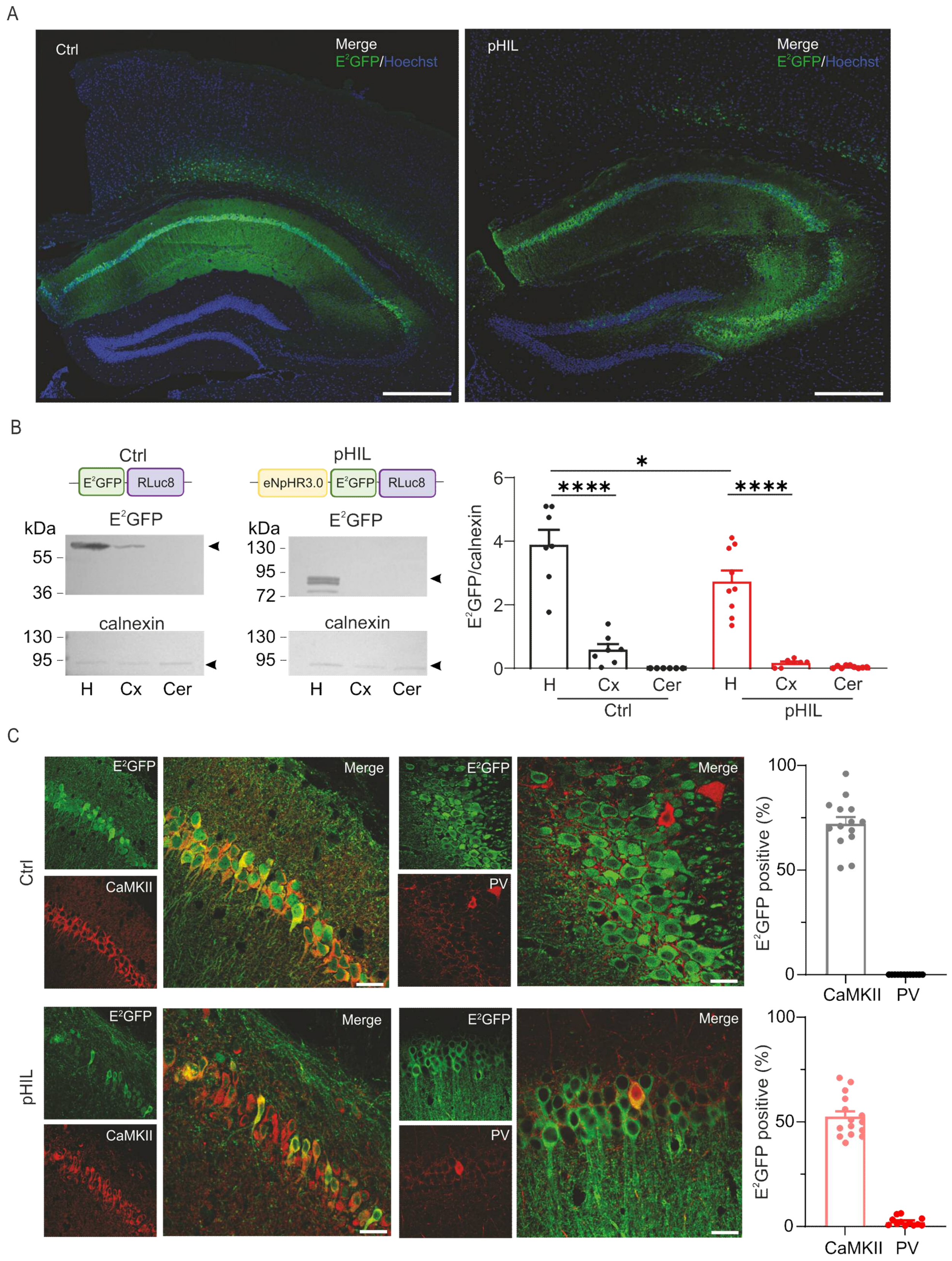


**Supplementary Figure 8. Cell-specific expression of pHIL in excitatory hippocampal neurons *in vivo*. A.** Representative fluorescence confocal images of brain slices from Ctrl- and pHIL-transduced mice labeled by the intrinsic $E^2$GFP fluorescence (green) and Hoechst to stain nuclei (blue) 1 month after the infection. Scale bar, 100 µm. **B.** Representative Western blotting showing Ctrl and pHIL expression in the cerebellum (Cer), cerebral cortex (Cx), and hippocampus (H). The expression of the fusion proteins was analyzed using anti-GFP antibodies with calnexin

immunoreactivity as loading control (*left*). The quantification of the expression in the three brain areas shows the successful transduction of the dorsal hippocampal region (n = 7, 9 mice for Ctrl and pHIL, respectively, one-way ANOVA $F(5,40)=43.41$ $p<0.0001$, Bonferroni's multiple comparison test *$p=0.0286$, ****$p<0.0001$). **C.** Confocal images of the hippocampus of mice transduced with either Ctrl (*upper row*) or pHIL (*lower row*) stained for excitatory (CaMKII; red) and inhibitory (PV; red) markers and by the intrinsic fluorescence of $E^2$GFP (green) present in both constructs. The representative images (*left*) and the quantification of the mean (± SEM) colocalization of $E^2$GFP signal with CaMKII and PV immunofluorescences (right) demonstrate the sole expression of the two probes in excitatory neurons (n = 14 fields of view from 3 mice per condition). Scale bar, 20 μm.

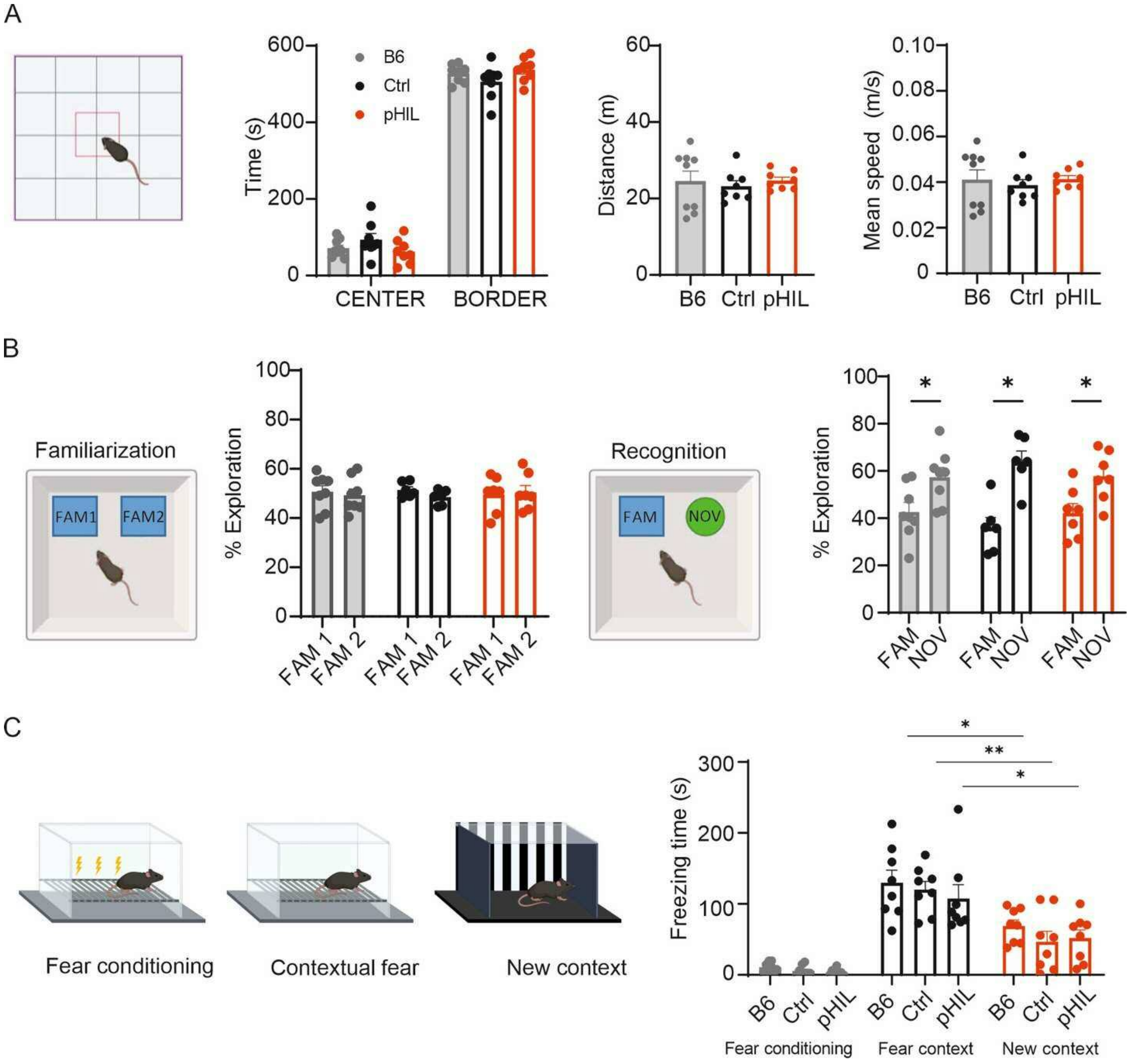


**Supplementary Figure 9. The *in vivo* expression of Ctrl and pHIL in the hippocampus does not alter the behavioral performances.** Behavioral tests were performed on Ctrl- and pHIL-transduced mice 4-5 weeks after infection, as well as on wild type C57BL/6 mice (B6). **A.** Open field test to evaluate anxiety levels through the analysis of the time spent in the border *versus* center, and motor performances evaluated as the total distance travelled and mean walking speed. No group differences in all parameters analyzed were found (n = 9, 8, 8 for B6, Ctrl and pHIL, respectively, two-way repeated measures ANOVA and one-way ANOVA). **B.** Novel object recognition test to evaluate cognitive abilities. No group differences in the percent time that mice spent to investigate the objects in the two test phases: the familiarization phase when mice were exposed to two identical objects and the recognition phase, when mice were exposed to one object previously explored and a novel unfamiliar object (n = 7, 6, 6 for B6, Ctrl and pHIL, respectively, two-sided paired Student's *t*-test *p=0.0156, *p=0.0236, *p=0.0258 for B6, Ctrl and pHIL, respectively). **C**. Contextual fear conditioning to assess hippocampal functions. No group differences in the freezing time analyzed during the conditioning phase, in the fear context and in the new context. All mice increased the time of freezing when located in the cage where they previously received the shocks (n = 8 for both B6, Ctrl and pHIL respectively, two-sided paired Student's *t*-test *p=0.0135, *p=0.026, **p=0.0016, *p=0.026 for B6, Ctrl and pHIL, respectively). Experimental setups drawings in panel A and B created

with BioRender.com released under a Creative Commons Attribution-NonCommercial-NoDerivs 4.0 International license (https://creativecommons.org/licenses/by-nc-nd/4.0/deed.en).

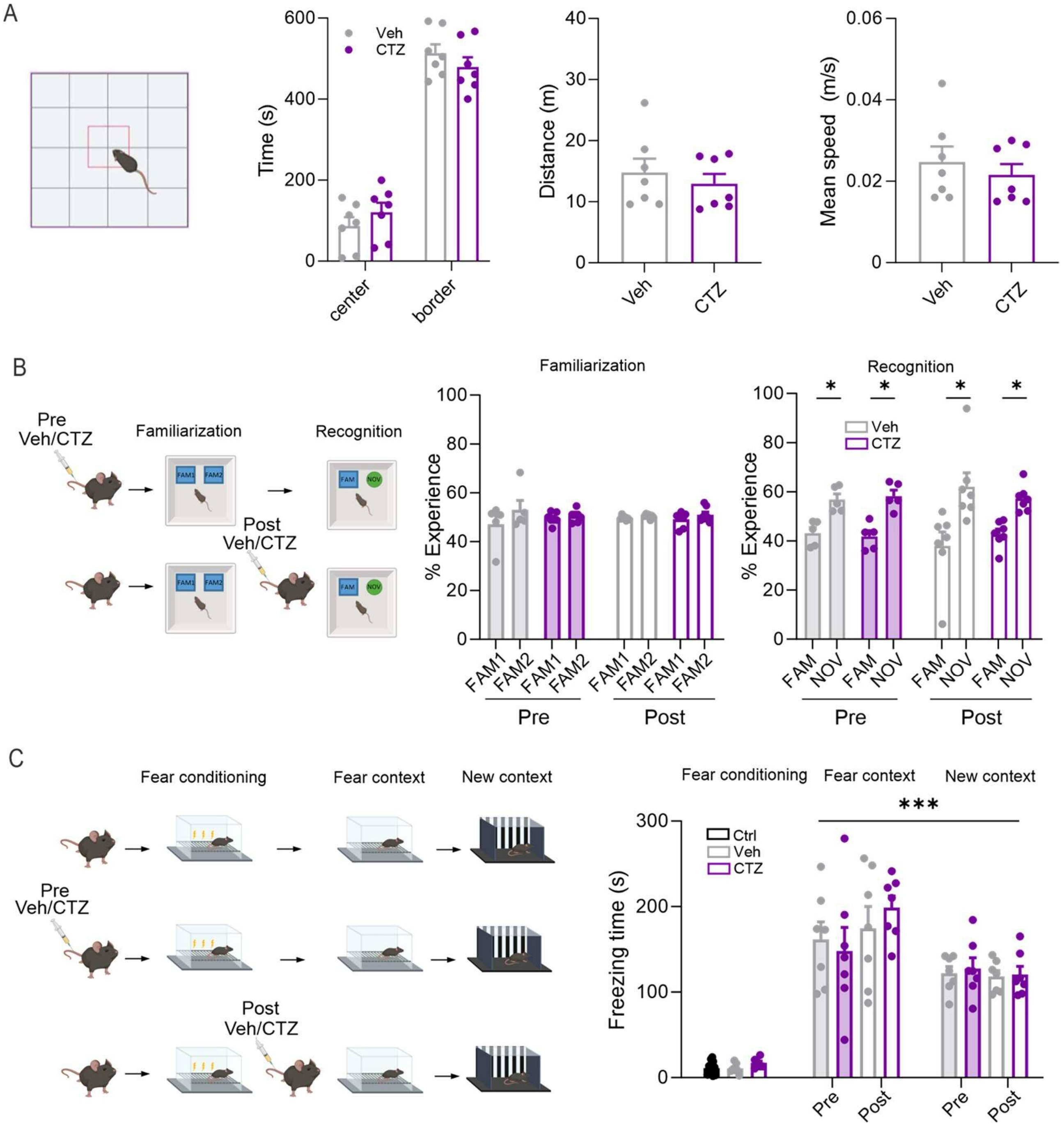


**Supplementary Figure 10. The activation of in vivo expressed pHIL by CTZ400a does not alter the behavioral performances.** Behavioral tests were performed on pHIL-transduced mice (4-5 weeks after transduction) following the administration of either CTZ 400a (CTZ; 0.3 mg/kg, i.v.) or vehicle. **A.** The time spent in the border *versus* center (*left*), and motor performances evaluated as the total distance travelled (*middle*) and mean walking speed (*right*) in the Open Field test are shown. No group differences in all parameters analyzed were found (n = 7 per experimental group). p>0.05, paired Student's *t*-test. **B.** Novel object recognition test to evaluate cognitive abilities. CTZ or the corresponding vehicle was administered either before the familiarization sessions or before the recognition test. The percent time that mice spent to investigate the objects was determined in both the familiarization phase (*left*) and the recognition phase (*right*) when mice were exposed to one object previously explored and a novel unfamiliar object. In the recognition test, all groups

significantly increased the exploration of the new object (two-sided paired Student's *t*-test/Wilcoxon test *p=0.0410, *p=0.0268, *p=0.0313, *p=0.0113 for Veh Pre, CTZ Pre, Veh Post, CTZ Post), but no significant group differences were observed (n = 7 per experimental group, two-way ANOVA). **C**. Contextual fear conditioning was performed to assess hippocampal learning and memory functions in pHIL-transduced mice (4-5 weeks after transduction). CTZ or the corresponding vehicle was administered either before the training fear conditioning sessions or before the fear context test. In the fear context test, all groups significantly increased their freezing behavior with respect to the new context. No group differences in the freezing times during the conditioning phase, in the fear context and in the new context were found (n = 7 per experimental group, two way ANOVA, group effect ns, trial effect $F(1,24)=19.18$ $p=0.0002$). For further details see Methods and legend to Supplementary Figure 10. Experimental setups in panel A, B and C drawings created with BioRender.com released under a Creative Commons Attribution-NonCommercial-NoDerivs 4.0 International license (https://creativecommons.org/licenses/by-nc-nd/4.0/deed.en).
