# Supplementary material for: A pH-sensitive closed-loop nanomachine to control hyperexcitability at the single neuron level
Source: Nat Commun. 2024 Jul 4;15:5609. doi: 10.1038/s41467-024-49941-3 (PMC11224301; doi:10.1038/s41467-024-49941-3)
Supplement: Supplementary file 3 — Description of Additional Supplementary Files [file 41467_2024_49941_MOESM3_ESM.pdf]

Description of Additional Supplementary Files

Supplementary Video 1. Pilocarpine (300 mg/kg; i.p.) triggered seizures in two representative wild type mice that had been transduced in the hippocampus with either Ctrl (left) or pHIL (right) and subjected to CTZ 400a (0.3 mg/kg, i.v.) injection immediately before pilocarpine.

Supplementary Video 2. Sound-evoked seizure responses of a representative PRRT2 KO mouse that had been transduced with pHIL in the hippocampus and assayed before (left) and after (right) the administration of CTZ 400a (0.3 mg/kg, i.v.).
